# Can dog-assisted and relaxation interventions boost spatial ability in children with and without special educational needs? A longitudinal, randomized controlled trial

**DOI:** 10.3389/fped.2022.886324

**Published:** 2022-07-29

**Authors:** Victoria L. Brelsford, Mirena Dimolareva, Elise Rowan, Nancy R. Gee, Kerstin Meints

**Affiliations:** ^1^School of Psychology, University of Lincoln, Lincoln, United Kingdom; ^2^School of Science, Department of Psychology, Bath Spa University, Bath, United Kingdom; ^3^Centre for Human-Animal Interaction, School of Medicine, Virginia Commonwealth University, Richmond, VA, United States

**Keywords:** animal-assisted intervention (AAI), dog-assisted, randomized controlled trial, longitudinal, child, special educational needs (SEN), cognition, spatial ability

## Abstract

Children's spatial cognition abilities are a vital part of their learning and cognitive development, and important for their problem-solving capabilities, the development of mathematical skills and progress in Science, Technology, Engineering and Maths (STEM) topics. As many children have difficulties with STEM topic areas, and as these topics have suffered a decline in uptake in students, it is worthwhile to find out how learning and performance can be enhanced at an early age. The current study is the first to investigate if dog-assisted and relaxation interventions can improve spatial abilities in school children. It makes a novel contribution to empirical research by measuring longitudinally if an Animal-Assisted Intervention (AAI) or relaxation intervention can boost children's development of spatial abilities. Randomized controlled trials were employed over time including dog intervention, relaxation intervention and no treatment control groups. Interventions were carried out over 4 weeks, twice a week for 20 min. Children were tested in mainstream schools (*N* = 105) and in special educational needs (SEN) schools (*N* = 64) before and after interventions, after 6 weeks, 6 months and 1 year. To assess intervention type and to provide advice for subsequent best practice recommendations, dog-assisted interventions were run as individual or small group interventions. Overall, children's spatial abilities improved over the year with highest increases in the first 4 months. In Study 1, typically developing children showed higher scores and more continuous learning overall compared to children with special educational needs. Children in the dog intervention group showed higher spatial ability scores immediately after interventions and after a further 6 weeks (short-term). Children in the relaxation group also showed improved scores short-term after relaxation intervention. In contrast, the no treatment control group did not improve significantly. No long-term effects were observed. Interestingly, no gender differences could be observed in mainstream school children's spatial skills. In study 2, children in SEN schools saw immediate improvements in spatial abilities after relaxation intervention sessions. No changes were seen after dog interventions or in the no treatment control group. Participants' pet ownership status did not have an effect in either cohort. These are the first findings showing that AAI and relaxation interventions benefit children's spatial abilities in varied educational settings. This research represents an original contribution to Developmental Psychology and to the field of Human-Animal Interaction (HAI) and is an important step towards further in-depth investigation of how AAI and relaxation interventions can help children achieve their learning potential, both in mainstream schools and in schools for SEN.

## Introduction

Children's visuospatial abilities are important in early development, and processing information about space is involved in infant's object location and locomotion (1). Spatial abilities develop gradually with age, and spatial reasoning encompasses the processing of space, shape, distance, direction, and angles, in addition to understanding these with reference to the self and the wider environment (1, 2). Children's egocentric representation (explaining the reference of objects relative to the self) gradually matures to include an allocentric representation (describing locations using external frames of reference such as objects relative to each other) (3–5). Accordingly, limits in performance on visuospatial tasks may therefore be due to the immaturity of neural networks involved in such functions (2).

Spatial cognition is intricately linked with problem-solving capabilities and high-level processing in the cognitive system (6). For example, spatial ability is associated with the development of mathematical skills in children (6–9) and plays a critical role in achievement in STEM topics (science, technology, engineering and mathematics) (10–12). Additionally, as spatial reasoning is part of humans' integrated neuro-cognitive system, wider functioning such as children's inhibitory control and attentional functioning are also likely to affect processing capabilities. For example, Beattie, Schutte, and Cortesa (13) found that children with better inhibitory and attentional ability had greater spatial working memory. These related abilities are integral to the learning process overall and affect academic performance.

It is noteworthy that spatial abilities may be influenced differently by the differing cognitive abilities in typically developing children and in those with special educational needs (SEN). For instance, children with Down Syndrome typically have a cognitive profile with impaired verbal processing abilities, but less impaired visuospatial processing abilities (14–18). Certain visuospatial abilities can also differ between those with Autism Spectrum Disorder (ASD) and Attention Deficit Hyperactivity Disorder (ADHD), and typically developing children (19, 20), for example in spatial perspective-taking (21) and other spatial tasks. Studies have reported that those with ASD can show superior ability in visuospatial processing tasks with particular strength in tasks focussed on detail and local feature processing, and poorer ability to attend to the spatial configuration as a whole, employing more global processing as detailed in Central Coherence Theory (22). However, these findings are not replicated in all circumstances-mixed evidence exists and more complex solutions, taking other cognitive processing function into account, are offered (23–26).

As with typically developing children, children with the same diagnosis vary in terms of their cognitive profile (23).

Additionally, the picture is more complex when taking gender differences in SEN populations into account as the over-representation of males makes the generalization of findings problematic (27).

Gender differences in spatial processing have been found in typical populations (28–30) with males often outperforming females (29, 31–34). Indeed Reilly, Neumann, and Andrews (35) suggest that of all cognitive functions, spatial processing shows the largest difference between genders. With its integral role for the development of quantitative reasoning skills important for mathematics and science subjects, this difference could contribute to gender differences in STEM subjects and underrepresentation of women in STEM fields. Theories have been offered to explain such differences on the basis of biological/hormonal factors (35–39), gender orientation and gender stereotypes [(35, 38), for wider discussion see (40)], socialization and play experience (35) and evolutionary pressures (41, 42). However, others argue that differences in spatial ability are often not present or are small (30, 40, 43, 44). Furthermore, evidence suggests that spatial skills are malleable, and can be improved through training (45–47), and that environmental factors and experience also play a large part in these observed differences (35, 48, 49).

As spatial processing and problem-solving are crucial to educational outcomes, for example in mathematics and in STEM subjects, it is important that such abilities are adequately supported in the school environment. This is especially pertinent as in recent years pupils' interest in STEM subjects, and uptake of STEM subjects by students, has dropped alarmingly (50).

One intervention which may enhance spatial abilities in children is an animal-assisted intervention (AAI). AAIs are becoming increasingly popular in educational settings as pets in the classroom may be beneficial to children's learning success (51), classroom behavior (52) and their emotional and cognitive development [see (53–57) for recent reviews and overview] as well as contribute to lower stress levels (58, 59). While animal-assisted interventions show some beneficial effects on human health and emotional well-being, learning and memory (55–57), it has been demonstrated that research in the field is growing, but also that the knowledge base still needs to be strengthened with many areas still under-investigated (60). In the past, research in this area has often suffered from small sample sizes, lack of control groups and overall insufficient scientific rigor (56, 61, 62). However, in recent years steady progress has been made in more thorough investigation of the effects of AAI on human health, well-being and cognition with improvements also found in executive function [e.g. (60, 63–65)].

Next to AAI, relaxation, meditation and yoga interventions have become increasingly popular in schools. They can help to improve mental and physical well-being, regulate stress, enhance performance on selective attention, concentration and mental flexibility tasks and psychomotor speed (66–74). Broderick and Metz (75) found that girls demonstrated increased feelings of calmness, relaxation and self-acceptance after mindfulness interventions. However, overall, the field is suffering from similar methodological problems as the earlier field of AAI.

Currently, only very few studies have been carried out on the effects of AAI on children's specific cognitive abilities. Previous studies have highlighted the beneficial effects of a dog's presence during a task on young children's cognitive functions such as memory (76), object recognition performance (77) and object categorisation tasks (78, 79). In addition, studies such as those of Hergovich, Monshi, Semmler and Zieglmayer (80) and Kotrschal and Ortbauer (52) reported increased classroom cohesion and improved behavior of children with a dog present which is an important factor in ensuring that conditions are optimal for learning. There are currently no studies investigating effects of dog-assisted interventions on children's cognitive development, and more specifically, there are no studies focusing on spatial abilities.

Explanations as to why dogs can have beneficial effects on humans are proposed by adapted and dynamic biopsychosocial models which integrate biological, physiological, psychological and social support (81–87), while others provide historical and social explanations [e.g., (88, 89)]. Physiological indices for arousal and affiliative behaviors have been identified as biological mechanisms underlying the human-animal bond (e.g., lower stress levels as indicated by lower cortisol, higher oxytocin levels, lowered blood pressure, reduced skin conductance, lower heart rate; (59, 90–93). Improved concentration, attention and motivation have been observed with the dog's presence creating a positive social atmosphere [for overview (90)]. Thus, an overarching, integrative approach combining neurobiological processes, attachment, biophilia and caregiving to pets may be best-suited to explain the resulting human-animal relationships, their development and their physiological and endocrine basis (83). Gee, Gryphon and McCardle (94) proposed a theoretical framework organizing the results of research and predicting unexplored pathways of indirect effects on learning through social-emotional development. This framework includes direct effects of classroom activities involving animals (mostly dogs) on children's motivation, engagement, self-regulation, and social interaction, as well as indirect effects on socio-emotional development and learning. This framework, though broadly useful, was not intended to serve as the basis for specific predictions within individual areas of cognitive development.

Despite spatial cognition being a crucial part of cognitive development and highly important to mathematics and STEM subjects, studies have so far not been carried out on the effects of animal-assisted interventions (AAI) or relaxation interventions on children's spatial cognition. The current study closes this knowledge gap and makes a novel and unique contribution to the field of animal-assisted and relaxation interventions within Developmental Psychology. We tested if dog-assisted interventions lead to enhanced spatial ability in children compared to relaxation interventions and compared to a no treatment control group.

Effects of AAI and relaxation interventions on children's spatial ability were investigated employing randomized controlled trials longitudinally, thus guaranteeing high scientific rigor. We tested typically developing children and children with special educational needs (SEN) to maximize knowledge gain in the field. Additionally, to provide practical advice for best practice in schools, intervention type was also assessed as to which works best [as the evidence is ambiguous (94)], and interventions were carried out as individual or small group interventions. This adds to the knowledge base as, depending on results, it may be possible to reduce direct contact time for therapy dogs per setting adding to animal welfare (95), and it may help to introduce the most cost-efficient intervention provision in educational settings (94).

In line with the above research, we predicted spatial ability improvements in the dog-assisted interventions compared to the control group when comparing pre- and post intervention periods. We expected intermediate effects for relaxation interventions and no or only maturational change in spatial abilities in the control condition. Concerning longer lasting effects, our longitudinal design allowed for exploration of such effects.

The current study was part of a larger, longitudinal, randomized controlled trial systematically examining the effects of dog- and relaxation-interventions on school children's academic performance, social and emotional well-being and measuring physiological changes (Lincoln Education Assistance with Dogs; https://lead.blogs.lincoln.ac.uk/) (95). The longitudinal studies described here investigated specifically the effects of AAI and relaxation interventions on spatial cognition in typically developing children (Study 1) and children with SEN (Study 2).

## Methods

### Participants

This research was approved by the University of Lincoln Research Ethics Committee (SOPREC) and is in line with British Psychological Society Ethics guidelines. In addition, the WALTHAM Animal Welfare and Ethical Review Board also approved the research.

Children were recruited through mainstream and special educational needs schools in Lincolnshire and Gloucestershire, UK. In Study 1, 105 children took part in Lincolnshire, UK (*N* = 54 males, 51 females, mean age = 8.91 yrs, SD = 0.39, min = 8.21, max = 10.07; 4 mainstream schools). In Study 2, 64 children (*N* = 54 males, 10 females, mean age = 9.27 yrs, SD = 0.79, min = 8.0, max = 11.5) from 7 SEN schools in Lincolnshire and Gloucestershire, UK, participated. Diagnoses for the latter included 15 children with ASD, 16 with ADHD, 12 with ASD and ADHD, 12 with learning disorder not otherwise specified (LD NOS), and 9 with unknown diagnoses as parents did not provide this information. Please see (Table 1) below for numbers of children taking part at each assessment point per condition and school type, and (Table 2) for retention rates and reasons for attrition.

**Table 1:** Number of children taking part in the test task at each assessment point per condition and school type.

**Cohort**	**Condition**	**Test 1**	**Test 2**	**Test 3**	**Test 4**	**Test 5**
		**(pre)**	**(post)**	**(6 weeks)**	**(6 months)**	**(1 year)**
		***N***	***N***	***N***	***N***	***N***
Special educational needs schools	Dog	26	26	25	16	17
	Relax	27	27	23	21	17
	Control	11	11	10	8	9
	**Total**	64	64	58	45	43
Mainstream schools	Dog	39	38	39	38	38
	Relax	39	38	39	36	37
	Control	27	27	27	26	26
	**Total**	105	103	105	100	101

**Table 2:** Retention rates and reasons for attrition per school type.

**Cohort**	**Retention rate**	**Reasons for attrition**
Mainstream schools	96.2 and 100%	Moving schools and illness absence
Special educational needs schools	67.18 to 100%	Moving schools, illness absence, appointments or increases in challenging behaviors preventing engagement with the task

All children attended school full-time. Researchers and dog handlers were in possession of enhanced police cheques, and researchers were highly experienced in research with school children.

### Dogs and handlers

Twenty-two different dogs and their handlers (*N* = 21) took part in the interventions on a volunteer basis. All handlers had insurance: *N* = 19 through their registration with Pets as Therapy, *N* =1 obtained separate insurance, and *N* = 1 was insured via their registration with Therapy Dogs Nationwide. All dog-handlers were required to attend safety training on understanding dog stress signaling behaviors before the study started. Dog breeds included: 1 Greek Hare-Hound, 2 Cavalier King Charles Spaniel and Miniature Poodle crossbreeds, 1 Labrador and miniature Poodle crossbreed, 2 German Short-Haired Pointers, 2 Miniature Schnauzers, 3 Labradors and 1 Labrador crossbreed, 2 Tibetan Mastiffs, 1 Border Terrier, 1 Scottish Terrier, 1 Lurcher, 1 Clumber Spaniel, 1 Yorkshire Terrier, 1 Pekingese, 1 Smooth Collie, 1 Cocker Spaniel and 1 Golden Retriever. All dogs were healthy and had been assessed by independent canine behavioral experts to ensure their suitability to work with children.

### Materials

The British Ability Scales (BAS-3) (96) were used to measure children's spatial ability (SA). The BAS-3 is a standardized cognitive scale normed for use from 5:00 to 17:11 (years: months) and designed to measure mental abilities significant for learning and educational performance (see https://www.gl-assessment.co.uk/assessments/products/bas3/ for more details). Two assessments within the BAS-3 were administered: Recognition of Designs and Pattern Construction to provide a Spatial Ability cluster score (SA). Children's performance in the Recognition of Designs task reflects their visual-spatial processing, short term visual memory, perception of spatial orientation and visualization abilities. Performance in the Pattern Construction task reflects the following: a child's visual-motor skills; spatial visualization (including matching abilities, perception of relative orientation, the ability to reproduce designs with objects, and to perceive and analyze visual information); non-verbal reasoning abilities (including skills in decomposing and reconstructing a design; the use of systematic strategies, for example, sequential assembly, hypothesis testing and trial and error); and the ability to follow verbal instructions and use verbal mediation strategies. After extensive assessment tool search and piloting, we chose the BAS-3 tool kit for the following reasons: it contained a range of assessments of the specific areas our research aimed to investigate, it was feasible to carry these out in realistic time slots with suitable duration for children of the chosen age group, it was usable and normed for both cohorts, and it was normed for British-English speaking children.

### Procedure

#### Informed consent

Parents gave consent for all children to take part in the study and provided details of any allergies and phobias to dogs. Children's assent was acquired prior to all test and intervention sessions, and parents and children were informed of their (and their children's) right to withdraw from the study at any time without having to give a reason. Dog handlers consented to taking part with their dogs and were free to withdraw at any time. Dogs were monitored continuously throughout the study for potential signs of wanting to withdraw. They also were free to retreat at any time.

#### Safety training and familiarization

All children took part in detailed safety training with dog body language training (95, 97) and further safety information before the study began, in order to set clear expectations for children's behavior around the dogs. This reduced the potential risk of incidents, and was designed to foster respect and uphold high standards of animal welfare. Children were familiarized with the dogs prior to study begin to eliminate potential novelty effects (95).

#### Testing

**Figure 1:**
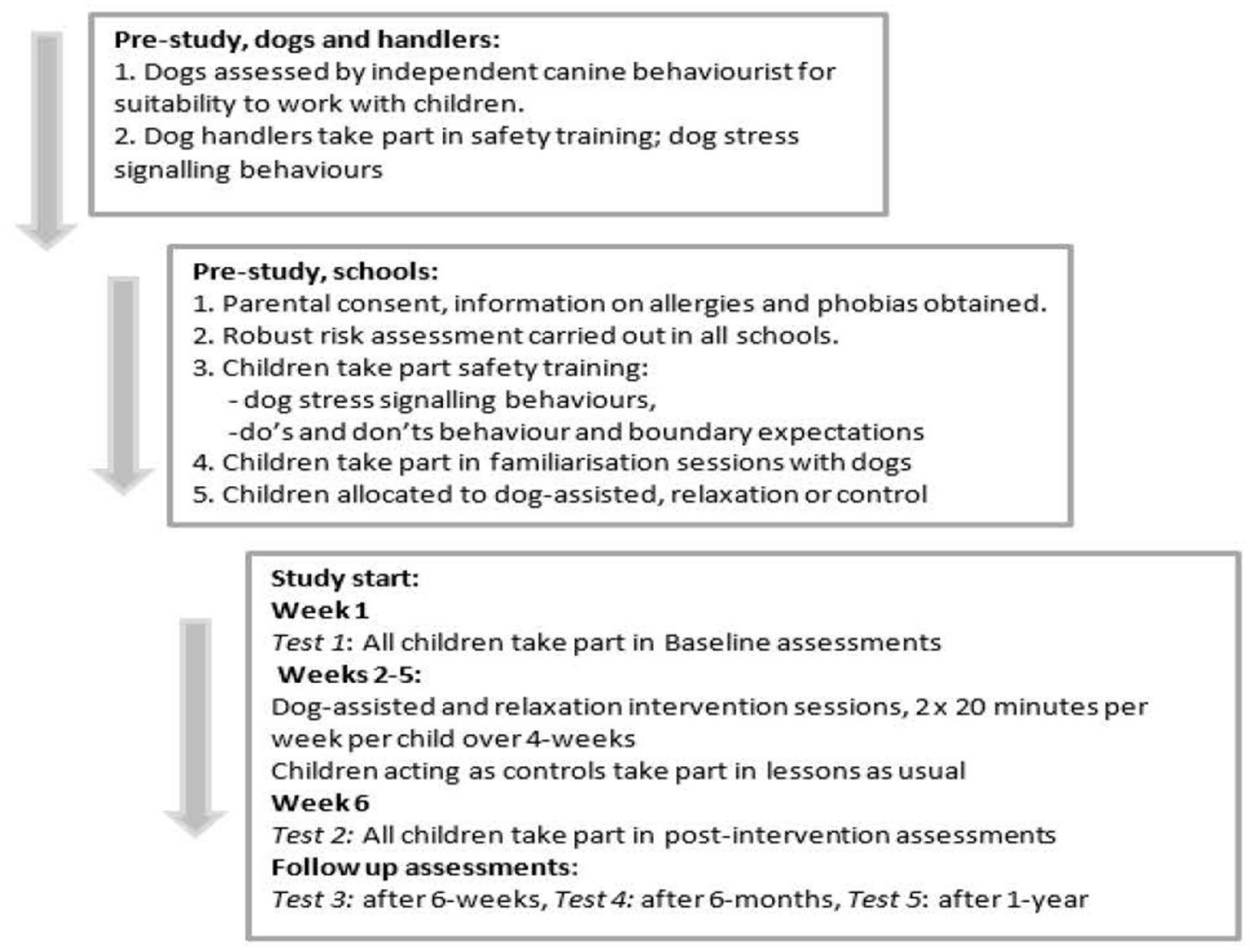
Welfare, safety, familiarization and consent and assent procedures carried out before and at study start for dogs and handlers, children, parents and schools taking part in the longitudinal randomized controlled trial.

Children's performance was assessed before interventions began (baseline), immediately after interventions, and was repeated after 6 weeks, 6 months and after 1 year to assess if interventions provided immediate, short-term, mid-term or long-term improvements to children's spatial ability. See Figure 1 below for overview of procedure details.

#### Interventions

Stratified randomization was used to place children in the different intervention groups. This method ensured that we did not confound dog ownership, socio-economic status or children's academic ability with intervention condition. Testing was carried out in schools in waves with 1/3 of participants in the dog group, 1/3 in the relaxation control group and 1/3 in the no treatment control group to avoid potential effects of seasonal affective disorder (SAD). For example, if the dog intervention would have taken place in summer, and the control groups in autumn or winter, we would have confounded the study and not been able to say if effects are due to SAD or our intervention conditions. Hence, to avoid confounding the study, all testing with all groups took place over the whole year as described above.

#### Individual and group interventions

Children were randomly assigned to take part either in individual or in small group interventions.

##### Dog-assisted intervention

Interventions took place in a separate room in schools during the normal school day. During the interventions, the researcher and the dog handler were present as were the dog and the children. Having completed all safety training, children were taken to the room, with the dog handler and dog waiting outside the room to greet the children (the dog had been familiarized to the room and with the children beforehand, see above). Children were asked to sit down and remain seated unless the activities taking place required them to do otherwise. Intervention sessions were 20 min and structured with approximately 5 min for initial dos and don'ts (e.g., “don't hug/kiss/crowd the dog,” etc.) and greeting the dog and handler. Then approximately 10 min were spent on learning facts about the dogs via the handler, talking about and interacting with the dog as deemed suitable by handler and researcher who were constantly observing the dog's signaling and body language in order to safeguard the dog's welfare. As all sessions were child-led, they varied somewhat in content. The last 5 min were spent saying goodbye to dog and handler and petting the dog as appropriate (again decided by dog handler and researcher).

##### Relaxation intervention

Relaxation sessions took part in a separate room and involved child age-appropriate meditation consisting of “Jellyfish” and “Butterfly” recordings from Enchanted Meditations for Kids (98) presented alternately across the sessions. Children were asked to lie down on a yoga mat and close their eyes; children who did not feel comfortable doing this, or who were unable due to mobility issues (mainly in SEN schools), were allowed to sit and relax with their eyes open or closed as they preferred. Again, the duration was 20 min, with timings approximately 5 min of active relaxation (body scanning with children moving toes, legs, fingers, etc.), followed by 10 min of meditation, and 5 min of active relaxation to match the profile of the dog sessions as closely as possible.

##### Control group

Children assigned to the no treatment control group condition took part in their usual class lessons.

#### Animal welfare considerations

A robust risk assessment was carried out for all settings taking part in the study (95). This incorporated strict protocols for animal welfare which were followed at all times. Care plans were completed for all dogs. Dogs were not required to work more than 2 h per day and had short breaks every 20 min as children moved between classrooms. Typical working times for most sessions were 1 h and 20 min in total. Dogs always had access to their own bedding for “time out” and water was freely available. Interventions would be stopped if dogs showed any signs of discomfort or being tired, and handlers were free to take their dog outside for a break as they felt appropriate. However, this did not occur.

#### Power calculation

Before study start, a priori power calculations were undertaken to determine sample size for the main repeated measures ANOVA with 3 conditions (dog intervention, relaxation intervention, and control group) and 5 measurement repetitions (GPower 3.1.9) (99); to obtain statistical power at the recommended 0.80 level for our analyzes (alpha at 0.05, for effect size up to 0.25), we required 27 children per cohort. For analysis including dog ownership, 42 participants were needed and for complex analysis with gender and dog ownership included (only possible in mainstream cohort due to expected typical male gender majority SEN samples), 60 participants were ideal. Due to the 5 repeated measures with the full study duration of 1 year, we overrecruited as feasible to avoid loss of power due to potential attrition.

#### Statistical analysis

Repeated measures ANOVAS were carried out overall, and for Study 1 (Mainstream school children) on Condition (dog intervention, relaxation intervention, no treatment control) and Time (before and after intervention, 6 weeks, 6 months, 1 year), also including Gender and Dog ownership for children. Analysis was then split into group and individual testing conditions. As we predicted a complex interaction pattern of improvements in spatial ability in children in the dog intervention group over the relaxation group, with no improvements expected in the no treatment control group, planned comparisons with Bonferroni corrections were calculated to investigate these specific predicted effects.

A similar pattern was followed for children in SEN schools (Study 2). However, due to the sample consisting mainly of boys, and due to missing information on dog ownership, we did not include Gender and Dog Ownership as factors in this analysis (see footnote, p. 12).

It is important to note that for all intervention conditions specific predictions, calculated with planned comparisons, were of core interest as it was predicted specifically that children in the dog intervention would show clear improvement after the intervention compared to the no treatment control group. Some improvement was expected in the relaxation group between pre and post intervention test times, and no significant improvement in the control group. Hence, planned comparisons were crucial to our analysis.

Significance testing follows the usual *p*-value criterion of smaller than 0.05 for significant results, and for planned comparisons smaller significance levels were used employing Bonferroni corrections. Statistical analysis was carried out using Statistica 12 as well as IBM SPSS, version 26. No data was excluded or replaced.

## Results

### Inspection of pre-intervention data for study 1 and study 2

Assumptions of normality were not violated for scores of spatial ability for either SEN schools: skewness of 0.678 (SE.297) and kurtosis of 1.482 (SE.586): Shapiro-Wilk (*W* = 0.966, *p* =0.075) or mainstream schools: skewness of 0.220 (SE 0.236) and kurtosis −0.031 (SE 0.467); Shapiro-Wilk (*W* = 0.992, *p* =0.797). Data from both cohorts were therefore analyzed using parametric analysis. Data were first assessed per cohort, then comparing cohorts. Within each analysis, results were first calculated for all children followed by analyzes for individual and group interventions.

### Initial comparison of cohorts

#### Assessment of baseline spatial ability

A one-way analysis of variance revealed that scores for spatial ability were significantly lower for children who attended SEN schools (*M* = 82.67) compared to those in mainstream schools (*M* = 95.30) [*F*_(1, 168)_ = 28.168, *p* < 0.001, $\eta_{\text{p}}^{2}$ =0.144]. A further one-way analysis to assess the effect of differing diagnosis on spatial ability within the SEN cohort showed no significant differences [*F*_(4, 59)_ = 1.094, *p* =0.368, $\eta_{\text{p}}^{2}$ =0.069] [ASD (*M* = 83.1); ADHD (*M* = 85.0); ASD-ADHD (*M* = 87.5); other (*M* = 77.0); unknown (*M* = 78.6)].

### Study 1: Effects of AAI on typically developing children's spatial ability (whole group)

A repeated measures ANOVA of Time (pre-intervention baseline, post-intervention, 6 weeks, 6 months, 1 year) x Condition (dog, relaxation, control) x Gender (male, female) x Dog ownership (dog, no dog) was conducted. Children showed a significant main effect of Time [*F*_(4, 336)_ = 32.358, *p* < 0.001, $\eta_{\text{p}}^{2}$ =0.278] with time accounting for a large amount of variance within the model. Spatial ability scores improved significantly over time overall, and most strikingly between the baseline and post-intervention [*t*_(102)_ = −5.070, *p* < 0.001, *d* = 0.49] and post-intervention and the 6-week assessments [*t*_(102)_ = −5.744, *p* < 0.001, *d* = 0.56]. No significant main effect for Condition [*F*_(2, 84)_ = 0.787, *p* = 0.459, $\eta_{\text{p}}^{2}$ = 0.018] occurred. No interaction for Time with Condition was revealed either [*F*_(8, 336)_ =0.728, *p* =0.667, $\eta_{\text{p}}^{2}$ =0.017].

To investigate our main question and the predicted effects of the dog and the relaxation intervention on spatial ability, planned comparisons with Bonferroni correction (*p* < 0.004) revealed that children in the dog interventions showed highly significant and immediate improvements in spatial ability from pre-intervention baseline to post-intervention (*t*_(37)_ = −3.499, *p* = 0.001, *d* = 0.56) and from post-intervention to 6-week testing [*t*_(37)_ = −4.507, *p* < 0.001, *d* =0.73], [*M* = 92.68 (baseline), 99.30 (post), 105.18 (6 weeks)].

Children who had taken part in the relaxation interventions showed no immediate, but significant short-term improvements in spatial ability scores from post-intervention to 6-week test times only [*t*_(37)_ = −3.861, *p* < 0.001, *d* =0.62], [*M* = 98.68 (post), 105.33 (6 weeks)]. The control group showed no significant improvements. No further significant main effects of Gender and Dog ownership, nor any interactions, were found (*p* < 0.05).

#### Individual intervention sessions

A repeated measures ANOVA of Time (pre-intervention baseline, post-intervention, 6 weeks, 6 months, 1 year) x Condition (dog, relaxation, control) x Gender (male, female) x Dog ownership (dog, no-dog) for individual intervention sessions showed a significant main effect of Time [*F*_(4, 204)_ = 21.436, *p* < 0.001, $\eta_{\text{p}}^{2}$ = 0.296], with children making significant improvements in spatial ability scores overall, and in particular from pre-intervention baseline to post-intervention [*t*_(67)_ = −4.575, *p* < 0.001, *d* =0.55] and post-intervention to 6-week tests [*t*_(67)_ = −3.784, *p* < 0.001, *d* =0.45]. There was no main effect for Condition [*F*_(2, 51)_ = 0.486, *p* = 0.618, $\eta_{\text{p}}^{2}$ = 0.019]. The interaction for Time with Condition [*F*_(8, 204)_ = 2.091, *p* = 0.038, $\eta_{\text{p}}^{2}$ = 0.076] reached significance and, in line with our predictions, further planned comparisons revealed that children in the individual dog interventions showed significantly improved scores {[*t*_(20)_ = −3.725, *p* = 0.002, *d* = 0.77]; (Bonferroni-corrected with *p* = 0.004) from pre-intervention baseline to post-intervention tests [*M* = 91.35 (pre), 101.30 (post)]}. Neither relaxation intervention [*t*_(20)_ = −1.428, *p* = 0.169, *d* = 0.31], nor the no treatment control group [*t*_(26)_ = −5.70, *p* = 0.010, *d* = 0.53] showed significant changes in scores immediately after intervention. No significant improvements in scores were visible from post intervention to week 6 for any of the groups. As above, no further significant main effects or interactions of Gender and Dog ownership were found.

#### Group intervention session

To assess the effects of AAI in group interventions, the same repeated measures ANOVA of Time (pre-intervention baseline, post-intervention, 6 weeks, 6 months, 1 year) x Condition (dog, relaxation, control) x Gender (male, female) x Dog ownership (dog, no-dog) was conducted. The significant overall effect of Time [*F*_(4, 184)_ = 20.726, *p* < 0.001, $\eta_{\text{p}}^{2}$ =0.311] was analyzed further and showed that children taking part in group interventions made significant improvements in spatial ability from baseline to post-intervention [*t*_(61)_ = −3571, *p* = 0.001, *d* = 0.45] and post-intervention to 6-week test times [*t*_(61)_ = −4.407, *p* < 0.001, *d* = 0.55]. No Condition main effect[*F*_(2, 46)_ = 1.238, *p* = 0.300, $\eta_{\text{p}}^{2}$ = 0.051] or interaction for Time with Condition reached significance [*F*_(8, 184)_ = 1.313, *p* = 0.239, $\eta_{\text{p}}^{2}$ = 0.054].

As above, we predicted specific improvements per condition, and planned comparisons revealed significant improvements in spatial ability for children in the group dog interventions. These occurred only from post-intervention to the 6-week test time [*t*_(17)_ = −3.713, *p* = 0.002, *d* = 0.87] [*M* = 97.06 (post), 104.58 (6 weeks)], i.e., not immediately following intervention (pre-intervention to post-intervention: *t* (17) = −1.113, *p* =0.281, *d* =0.26), but somewhat delayed compared to the individual interventions.

No significant relaxation effects were seen pre to post (*t* (16) = −2.124, *p* =0.050, *d* =0.51) or post to 6-weeks (*t* (16) = −2.880, *p* =0.011, *d* =0.69). The no treatment contros group also failed to show significant improvements (pre-post (*t* (26) = −2.794, *p* =0.010, *d* =0.53); post to 6-weeks (*t* (26) = −1.535, *p* =0.137, *d* =0.29). There were no further significant main effects or interactions of Gender and Dog ownership.

### Study 2: Effects of AAI on children's spatial ability in SEN schools (whole group)

A repeated measures ANOVA of Time (pre-intervention baseline, post-intervention, 6 weeks, 6 months, 1 year) x Condition (dog, relaxation, control) was conducted^1^. A significant main effect of Time demonstrated SEN school children's significant improvements in spatial ability scores [*F*_(4, 128)_ = 4.926, *p* = 0.001, $\eta_{\text{p}}^{2}$ = 0.133) with time accounting for a large amount of variance within the model. Significant improvements occurred from baseline to post-intervention test times (*t*_(632)_ = −4.577, *p* < 0.001, *d* = 0.57). No differences were found between other consecutive test times across the length of the study. As expected, also for this cohort, there was no significant main effect for Condition [*F*_(2, 32)_ = 0.149, *p* = 0.862, $\eta_{\text{p}}^{2}$ =0.009] and no interaction with Time [*F*_(8, 128)_ = 0.312, *p* = 0.960, $\eta_{\text{p}}^{2}$ = 0.019].

As with the typical cohort, planned comparisons were calculated to find out if interventions had an effect on spatial ability (Bonferroni significance level: *p* < 0.004). Only children in the relaxation condition [*t*_(26)_ = −3.521, *p* = 0.002, *d* = 0.67) made significant improvements from pre-intervention baseline to post-intervention assessments [*M*= 81.19 (baseline), 87.67 (post)]. However, children in the dog [*t*_(25)_ = −2.654, *p* = 0.014, *d* = 0.52] and control conditions [*t*_(10)_ = −1.314, *p* = 0.218, *d* = 0.39] did not improve their scores significantly.

#### Individual intervention session

For individual interventions a repeated measures ANOVA of Time (pre-intervention baseline, post-intervention, 6 weeks, 6 months, 1 year) x Condition (dog, relaxation, control) only showed a significant main effect of Time [*F*_(4, 60)_ = 3.891, *p* = 0.007, $\eta_{\text{p}}^{2}$ = 0.206], with children with special educational needs significantly improving from baseline to post-intervention test times only [*t*_(25)_ = −3.840, *p* = 0.001, *d* = 0.75]. No main effect for Condition [*F*_(2, 15)_ = 0.935, *p* = 0.414, $\eta_{\text{p}}^{2}$ = 0.111] or interaction for Time with Condition reached significance [*F*_(8, 60)_ = 0.605, *p* = 0.770, $\eta_{\text{p}}^{2}$ = 0.075].

To investigate the predicted intervention effects, planned comparisons showed that scores for children did not increase significantly between the pre-and post-intervention {dog [*t*_(6)_ = −1.837, *p* = 0.116, *d* = 0.69], relaxation [*t*_(7)_ = −4.080, *p* = 0.005, *d* = 1.44], control [*t*_(10)_ = −1.314, *p* = 0.218, *d* = 0.39] with the relaxation group just missing significance (Bonferroni-adjusted significance level: *p* = 0.004)}.

No significant increases in scores were found either from post-intervention to 6-week test times {dog [t_(6)_ = −1.276, *p* = 0.249, *d* = 0.48], relaxation [*t*_(4)_ = 0.607, *p* = 0.577, *d* = 0.27], control [*t*_(9)_ = −1.664, *p* = 0.130, *d* = 0.52]. No other comparisons reached significance}.

#### Group intervention session

**Figure 2:**
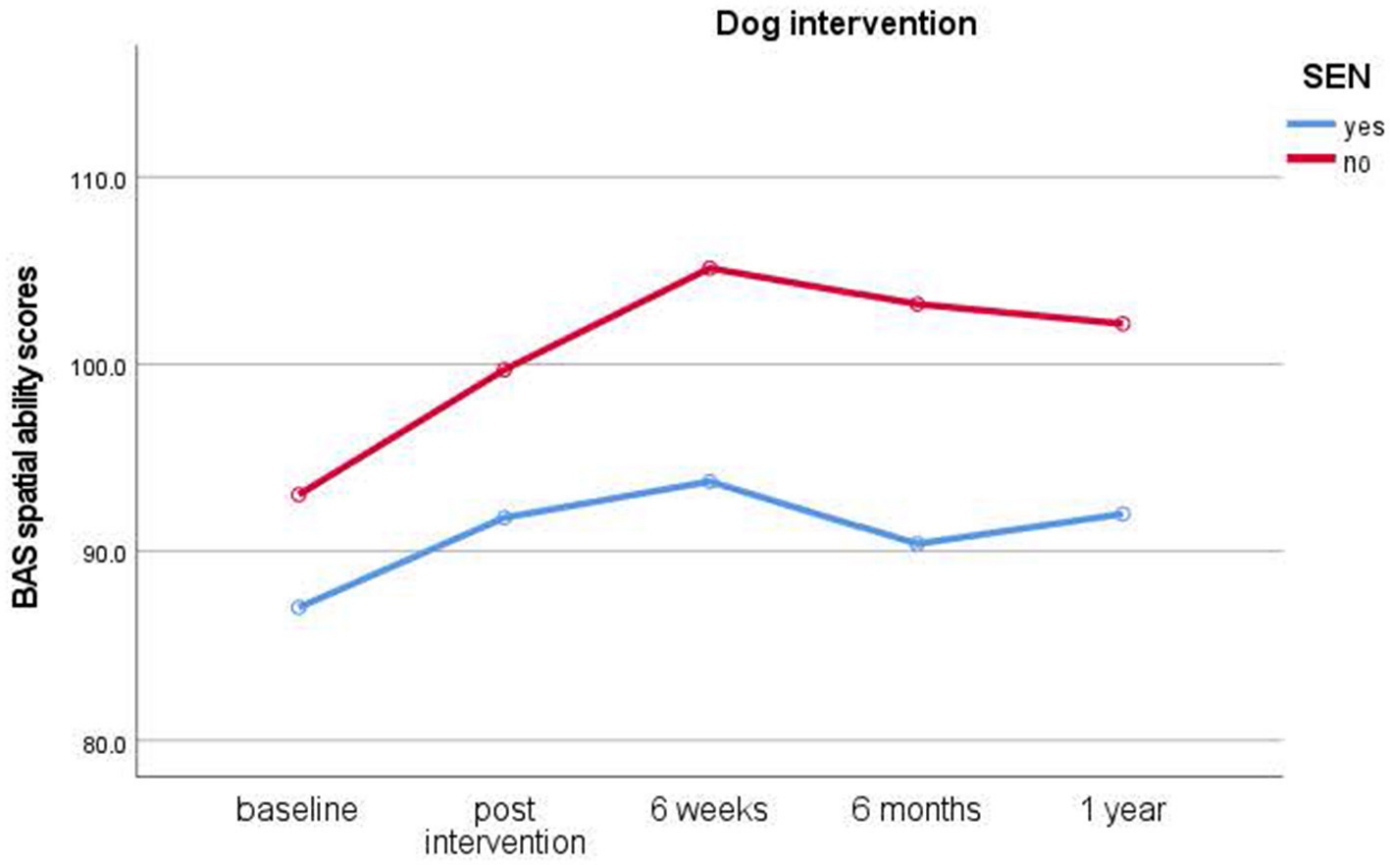
Results of longitudinal assessments in the dog intervention: means for British Ability Scale spatial ability scores (y-axis) over time (x-axis) in the dog intervention group for children with and without special educational needs (SEN). Higher scores imply higher ability.

**Figure 3:**
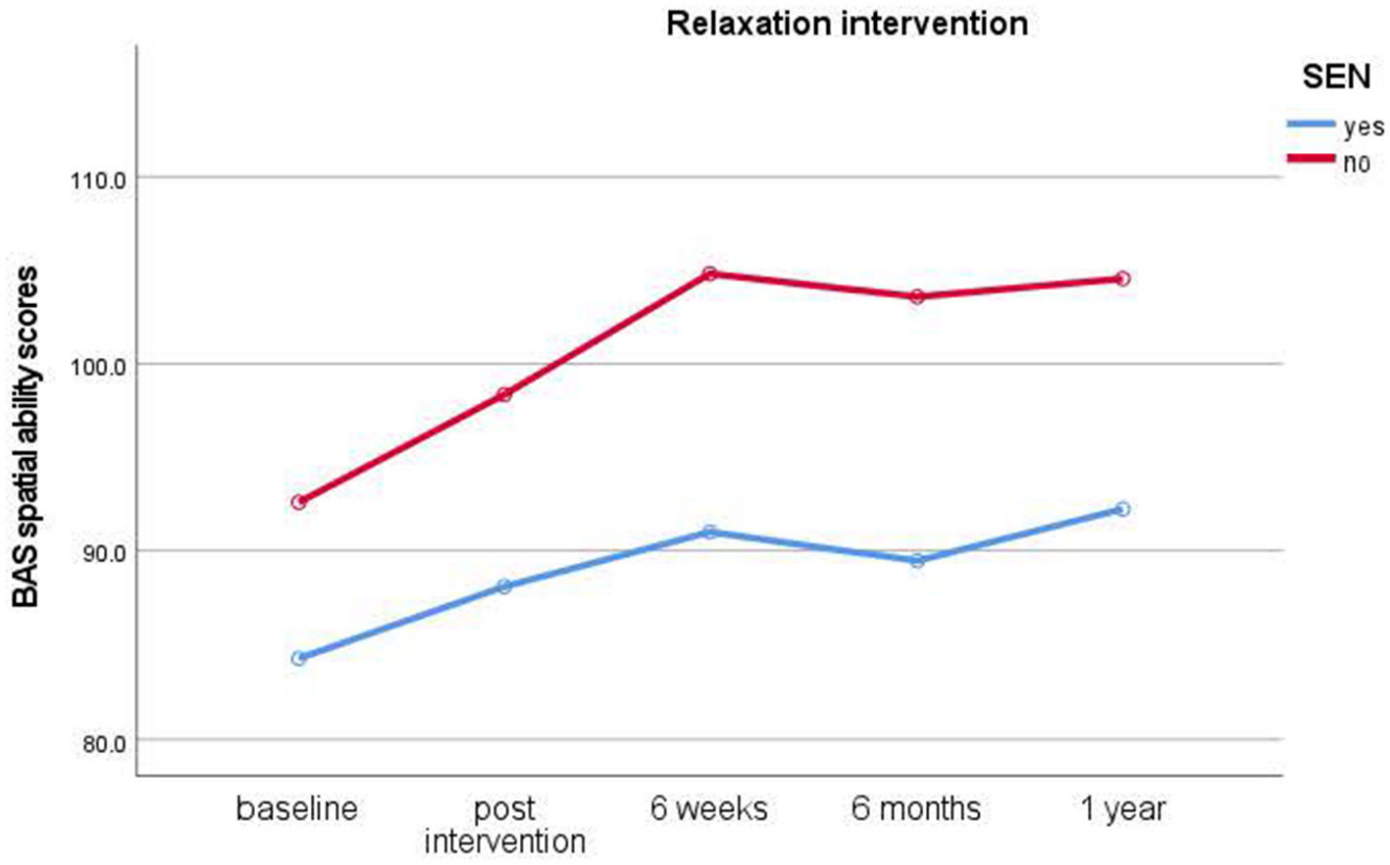
Results of longitudinal assessments in the relaxation intervention: means for British Ability Scale spatial ability scores (y-axis) over time (x-axis) in the relaxation intervention group for children with and without special educational needs (SEN). Higher scores imply higher ability.

**Figure 4:**
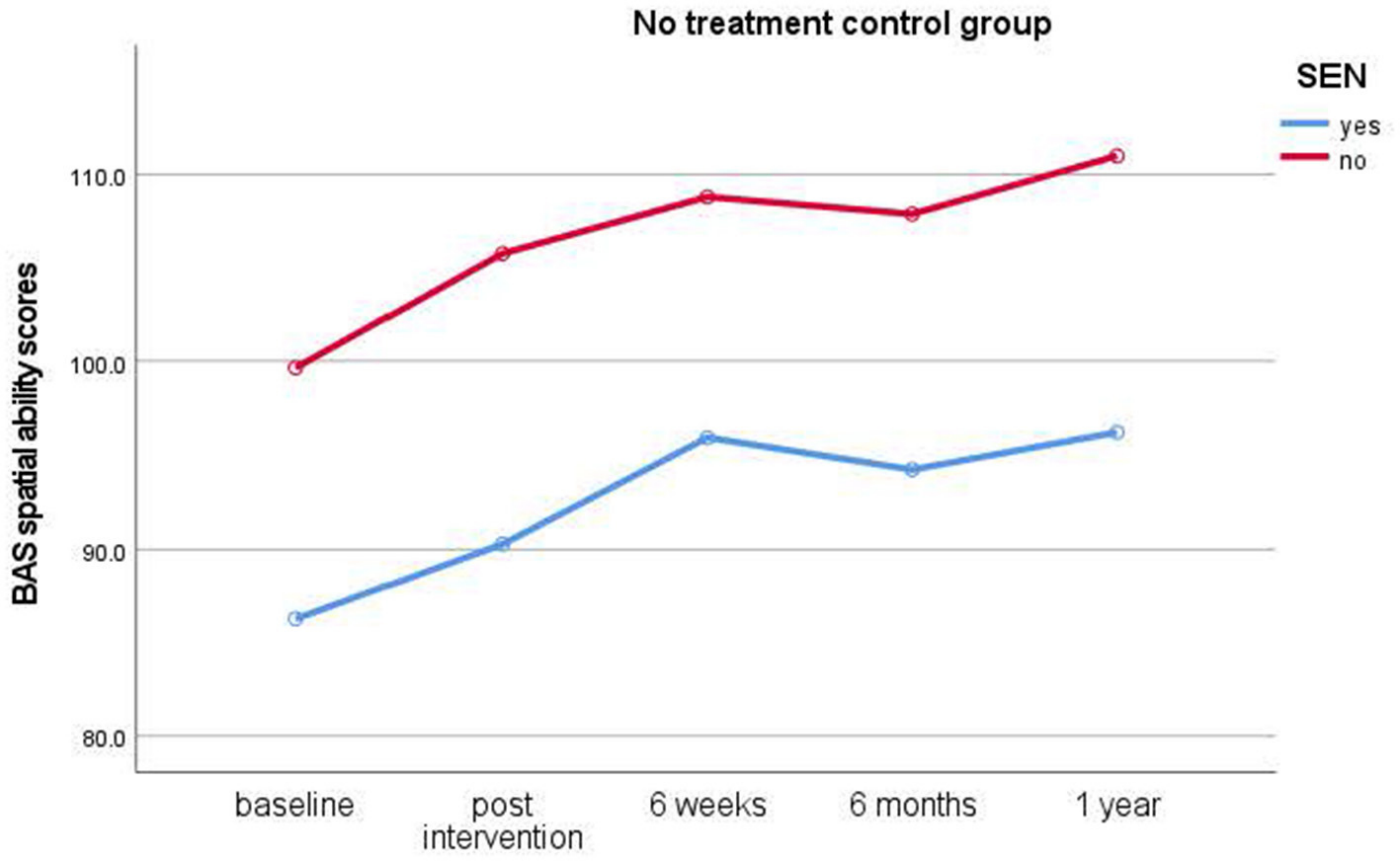
Results of longitudinal assessments in the no treatment control group: means for British Ability Scale spatial ability scores (y-axis) over time (x-axis) in the no treatment control group for children with and without special educational needs (SEN). Higher scores imply higher ability.

To assess results for children who took part in group interventions, the repeated measures ANOVA of Time (pre-intervention baseline, post-intervention, 6 weeks, 6 months, 1 year) x Condition (dog, relaxation, control) revealed a significant main effect of Time [*F*_(4, 84)_ = 3.231, *p* = 0.016, $\eta_{\text{p}}^{2}$ = 0.133). Children taking part in group interventions made significant improvements in spatial ability from baseline to post-intervention test times [*t*_(48)_ = −3.092, *p* = 0.003, *d* = 0.44]. No significant Condition main effect [*F*_(2, 21)_ = 0.493, *p* = 0.617, $\eta_{\text{p}}^{2}$ = 0.045] or interaction for Time with Condition occurred [*F*_(8, 84)_ = 0.325, *p* = 0.954, $\eta_{\text{p}}^{2}$ = 0.030]. Planned comparisons investigating the predicted intervention effects showed that children did not improve significantly from pre to post-intervention {dog [*t*_(18)_ = −1.998, *p* = 0.061, *d* = 0.45], relaxation [*t*_(18)_ = −1.882, *p* = 0.076, *d* = 0.43], control [*t*_(10)_ = −1.314, *p* = 0.218, *d* = 0.39]} and post-intervention to 6-week test times {dog [t_(17)_ = 0.170, *p* = 0.897, *d* = 0.04), relaxation [*t*_(17)_ = −1.239, *p* = 0.232, *d* = 0.29], control [*t*_(9)_ = −1.664, *p* = 0.130, *d* = 0.52]}. No other consecutive tests were significant. Figures 2–4 below illustrate in overview the main results.

## Discussion

Children's spatial abilities are a crucial part of their learning and cognitive development, and important for children's problem-solving capabilities, the development of mathematical skills and progress in STEM topic areas. As many children, with and without SEN, struggle with maths and STEM topics, and as these topics have suffered a significant loss of interest by school children and decline in uptake in students (50), it is worthwhile to study how learning and performance can be enhanced at an early age.

This study is the first to investigate if dog-assisted and relaxation interventions can improve spatial abilities in school children. The study employed high scientific rigor by using randomized controlled trials and a longitudinal design. We also broadened the scope of the research by including both children attending mainstream and special educational needs schools (SEN). As it has been hitherto unknown if individual or group interventions work better, the study also assessed the effects of individual and group interventions to make recommendations for best and most efficient practice.

The results outline how typically developing children and children with SEN developed over the year during which time all children's spatial ability scores increased significantly from baseline over the 1-year study duration and thus showing the expected general learning and maturation effects. Immediate and short-term improvements were also revealed after 4-week interventions.

Study 1 results indicate that typically developing children benefitted from the dog intervention. Improvements in spatial ability scores occurred immediately after the intervention and lasted up to 6 weeks, with effect sizes ranging from medium to large. Interestingly, individual dog interventions showed more immediate effects, while group interventions had somewhat delayed effects with children showing better scores after intervention end to 6 weeks. Likewise, children in mainstream schools who took part in the relaxation intervention also benefitted from these overall, albeit relaxation interventions showing no immediate, but significant short-term improvements in spatial ability scores from post-intervention to 6-week test times. In contrast, it is noteworthy that no significant improvements in spatial ability scores were seen in the no treatment control group.

Overall, the results show that dog and relaxation interventions enhance mainstream school children's spatial abilities, and it noteworthy to point out that the dog intervention shows significant results throughout, with individual sessions having a more immediate effect and group sessions a delayed effect. It could be argued that individual sessions involved more intensive interaction between children and dogs and therefore stronger calming effects in line with Beetz and colleagues (90). This may have had a beneficial effect on children's processing of spatial tasks shortly after the interventions. Children in the group sessions had less intensive contact time with the dogs, but they had instead other group members to share the experience with which could contribute to a delayed effect. Future research will need to establish if the less intense animal experience combined with peer contact and potential later discussions may have led to a delayed beneficial effect.

Study 2 revealed, in contrast to the typically developing cohort, that children with special educational needs (SEN) showed a significant increase in spatial ability in the relaxation condition only. They showed significant improvements from baseline to post-intervention assessments with medium effect sizes. While children in the dog condition also showed improved scores, these differences did not reach significance. Likewise, children in the no treatment control condition also did not show a significant improvement in scores. In the SEN cohort, no clear advantage for either individual or group interventions became evident from the data. Thus, this cohort benefitted from relaxation interventions instead of dog-assisted interventions.

The integrative dynamic biopsychosocial model (82, 83) is best suited to explain the result patterns for both cohorts, based on the stress-reducing and calming effects of both interventions, including the creation of a positive atmosphere, beneficial to learning, in dog-assisted interventions and relaxation interventions (82, 83, 85, 86, 89, 90, 92). Concerning specifically spatial ability tasks it should be highlighted that these involve working memory, which incorporates integrated systems of the central executive, phonological loop and visual-spatial sketchpad (100). These flexible integrated cognitive systems can be affected by individual factors and wider influences such as learning, emotion and stress (101). Previous AAI research has shown positive effects on memory during cognitive tasks (76–79, 102). Positive emotions can also have a beneficial effect on spatial working memory (103–106), and affective states can influence working memory (101). Relaxation and stress reduction as shown in other research on cortisol level buffering in AAI is likely involved during both dog and relaxation interventions (58, 59), and may have benefitted the spatial ability tasks. Likewise, improvements in executive functioning, which have been linked to the presence of a dog in college students (63) and school children (64), may be driving improvements in spatial ability scores. As one is able to inhibit irrelevant thoughts, relax and focus on the task at hand, general cognitive abilities, such as spatial ability, also improve.

Regarding the developmental pattern over the year, it is noteworthy that children's scores did not rise as steeply (or significantly) after the 6-week follow up point. This may be as children's cognitive scores may fluctuate as the school term progresses, as learning and development do not always represent a linear process (105, 106).

Potentially, the repeated use of the BAS tool kit could present a limitation of the current study in case the closer test intervals at the beginning of the study (baseline / after the 4-week intervention / 6 weeks later) may have resulted in practice effects enhancing the test results up to the 6-week time point, and which may dissipate after a longer break of 6-months. However, it is unclear how likely this scenario is given the complexity of the tasks and given the differences in results in the experimental groups and the no treatment control group. Further studies may also include a different, or a combination of, cognitive instruments. In the current study we were limited to choose one cognitive assessment tool due to other measures taking place within the overall larger-scale project as mentioned above.

The lack of further significant improvements suggests that dog interventions may not show longevity past the post-intervention test time or the later assessment 6 weeks after post intervention testing (in week 12).

Concerning cohort differences, children's scores of spatial reasoning were significantly higher for those attending mainstream schools than for those with special educational needs (2, 19, 20). This is in line with previous research showing differing performance in children with neurotypical and non-neurotypical developmental profiles. Within the SEN cohort, processing of spatial ability was not significantly different based on the diagnoses of the children. This is a noteworthy finding, given that different diagnoses have diverse aetiologies and so differ in terms of their neural systems, memory, attention and executive function which are integral to efficient visuospatial processing. The current results are therefore consistent with those studies that did not find superior ability in visuospatial processing tasks in participants with ASD (24–26). As spatial reasoning is important in many other areas of learning such as the STEM topics and is malleable (46) it would be interesting and worthwhile to assess whether AAI paired with specific skills training can foster long term benefits for children's spatial ability.

As pet ownership may have additional beneficial effects on the health and well being of children (107), and as it is unknown how this may interact with the effects of AAI and relaxation interventions, dog ownership was included in this longitudinal study. However, no effects of dog ownership were found, nor were there any interactions. This finding suggests that for populations (typical and SEN) of 8–10-year-old children, dog ownership is not necessary for the accrual of benefits from interacting with a therapy dog in interventions. It may be useful in future to investigate attachment to pets and attitude to pet dogs to find out if this potentially influences interaction outcomes.

With interventions taking place twice a week over 4 weeks, and the cognitive assessments carried out without the presence of a dog in the room, the current study adds to previous research showing beneficial effects on cognitive tasks with a dog present during testing [see (76–79)]. As there are no comparable studies into the effects of AAI on children's spatial cognition over time (56), the current research pioneers longitudinal investigation of AAI and relaxation interventions.

Interestingly, despite previous research and theories reporting gender differences in spatial skills, this study found no significant differences between girls and boys on the standardized tests. These results are in line with research showing small or no differences (30, 42–44, 108) and potentially highlight the influence of teaching [e.g., (35, 47)].

While individual intervention sessions require more working hours with dogs and handlers, group sessions could mean cost efficiencies for schools and reduced working time for therapy dogs. However, the results of the current study indicate that the individual dog interventions may be more effective. To our knowledge, there are no systematic studies on how dosage of interventions may relate to intervention type (individual or group) – future research should be carried out to enable effective interventions.

Concerning potential feasibility, organizational, ethical and safety challenges in school settings, the following should be highlighted: For this longitudinal, randomized controlled trial in schools with two child cohorts to be feasible, it required early and meticulous planning. Next to the usual complex planning involved in longitudinal studies, further protocols concerning ethics and safety had to be established and implemented, including, for example, school, parent and child consent/assent. We operated with a timetable that was agreed in advance with schools and dog handlers and we managed to maintain schools' and children's continued interest and cooperation. Concerning human and animal safety and welfare, we have successfully employed the Lincoln Education Assistance with Dogs (LEAD) risk assessment tool (95) for this study – the tool not just ensured a thorough risk assessment, and provided a structure with clear areas of responsibility, but also enabled consistent, safe and welfare-guided practice for all involved.

We would therefore recommend the following steps as vital for successful AAI and AAI research in schools:

(1) Timing and Commitment: Following appropriate ethics approval, ensure significant advance recruitment of schools with clear information as to what the requirements are concerning time and space (e.g., separate room for specific duration). It is useful to be clear about the amount of commitment needed from schools and teachers so all involved can agree to researchers spending a substantial amount of time in schools with the children.
(2) Clarity of Information: Transparency concerning the study to inform teachers, parents and children of all that is involved is essential to obtain consent/assent as well as to maintain ongoing interest.
(3) Safety and welfare: Human and animal safety and welfare need to be ensured at all times. The LEAD tool (95) for AAIs as well as safety training for all involved as described above [e.g., on dog body language (97)] is efficient and helps to raise awareness of potential risk and ensure the safety and welfare of all involved.

In conclusion, this longitudinal RCT study is the first to demonstrate how children's spatial abilities can benefit from AAI with dogs and from relaxation interventions. In Study 1, typically developing children showed improvements in spatial abilities especially over the first 12 weeks, but also beyond, and those in the dog group showed significant improvement immediately after the intervention and also short-term (a further 6 weeks after intervention end). They also showed significantly enhanced performance short-term after relaxation interventions. In contrast, no significant improvements in spatial abilities were found in the no treatment control group.

In Study 2, the cohort of children with SEN showed lower scores overall, showed most learning only in the first 6 weeks, and benefitted only from relaxation interventions. Intervention effects did not extend to the second testing point after the end of the intervention.

As immediate and short-term effects, but not long-term effects were evident, and as spatial abilities are important for wider academic skills such as maths and STEM topics in both cohorts, it is recommended that further research assesses how AAI and relaxation interventions may be incorporated into training applications to enhance such skills. Furthermore, we need to understand better why a dog intervention may improve these skills in typical children, but not in SEN children. It is possible that with SEN children a longer or more intensive period of intervention (higher dosage) may be required to accrue benefits, if any, of an AAI.

This study provides information about the time course of effects of one type of AAI on spatial ability, but many variables need to be examined in the future such as dosage of intervention (number of days of AAI per week, number of weeks of AAI), details of the intervention (do the children need to touch the dog), and delivery of the intervention (free form vs planned pedagogy). The underlying mechanisms of action and the potential for interaction among these mechanisms need to be investigated in further depth in future so that we may make effective recommendations for the use of AAI in typical and SEN children in future.

## Data availability statement

The raw data supporting the conclusions of this article will be made available by the authors, without undue reservation.

## Ethics statement

The studies involving human participants were reviewed and approved by University of Lincoln Psychology Research Ethics Committee (SOPREC) and also WALTHAM Animal Welfare and Ethical Review Board. Written informed consent to participate in this study was provided by the participants' legal guardian/next of kin.

## Author contributions

KM conceived the study and obtained the research funding. VB contributed to conception of the project. KM, NG, and ER advised on data collection and analysis. VB and MD collected the data. ER oversaw the data base. VB, MD, KM, and ER collated and/or analyzed the data. All authors contributed to the final research design of the current study. All authors contributed to advice on analysis and to writing the manuscript, and read and approved the final manuscript.

## Funding

This research was funded with a research grant from the WALTHAM Petcare Science Institute (formerly Waltham Centre for Pet Nutrition), Mars Petcare, and a grant from the Waltham Foundation.

## Conflict of interest

The authors declare that the research was conducted in the absence of any commercial or financial relationships that could be construed as a potential conflict of interest.

## Publisher's note

All claims expressed in this article are solely those of the authors and do not necessarily represent those of their affiliated organizations, or those of the publisher, the editors and the reviewers. Any product that may be evaluated in this article, or claim that may be made by its manufacturer, is not guaranteed or endorsed by the publisher.

## Footnotes

^1^The majority of SEN children were boys, hence, we could not include Gender in our analysis. Information on Dog Ownership for this cohort was incomplete due to parental non-responses. Due to expected higher attrition in this group, we calculated the power a priori: with assumed power of 0.8, 3 groups and 5 measurement repetitions, a sample size of 27 is required (significance level at 0.05, effect size 0.25).
